# Moral intensity and ethical sensitivity in pre-service therapeutic sciences clinicians: a vignette-level analysis of ethical principle recognition and ethical sensitivity skills

**DOI:** 10.1186/s12910-026-01521-4

**Published:** 2026-06-12

**Authors:** Alida Naudé, Juan Bornman

**Affiliations:** Department of Health and Rehabilitation Sciences, Division of Speech-Language and Hearing Therapy, Stellenbosch University, Tygerberg Campus, Cape Town, South Africa

**Keywords:** Ethical sensitivity, Moral intensity, Healthcare education, Ethics training, Therapeutic sciences, Ethical decision-making

## Abstract

Ethical sensitivity is a foundational component of moral functioning, enabling individuals to recognise ethically salient aspects of situations. In healthcare education, this capacity is critical for ethical practice. While ethics education often emphasises ethical principle knowledge, less is known about how contextual features of moral situations—such as moral intensity—relate to different components of ethical sensitivity. This retrospective cross-sectional study examined vignette-level associations between moral intensity and ethical sensitivity using previously collected data from 120 final-year pre-service clinicians across the therapeutic sciences. Twelve clinical vignettes from the Measuring Instrument for Ethical Sensitivity in the Therapeutic Sciences (MIEST) were independently rated for moral intensity using Jones’s six-dimension framework. Moral intensity scores ranged from 20 to 28 (possible range 6–30). Moral intensity was not associated with ethical principle recognition or overall ethical sensitivity scores. However, a strong positive vignette-level correlation was observed between moral intensity and ethical sensitivity skill identification (*r* = 0.81, *p* = 0.001). These findings provide preliminary empirical support for ethical sensitivity as a multidimensional construct and suggest that contextual features of moral situations may differentially engage perceptual–affective components of ethical sensitivity.

**Supplementary Information:**

The online version contains supplementary material available at 10.1186/s12910-026-01521-4.

## Background

Ethical sensitivity, defined as the ability to perceive, recognize, and accurately interpret ethically salient features of clinical situations, constitutes the foundational component of ethical decision-making in healthcare. Ethical sensitivity is positioned as the essential first step in Rest’s Four-Component Model; individuals cannot engage in ethical reasoning about issues they fail to recognize [1]. This perceptual capacity has gained increasing recognition as critical to ethical practice, particularly in professions requiring autonomous clinical decision-making with vulnerable populations [2]. Recent advances in healthcare ethics education have highlighted the multidimensional nature of ethical sensitivity, encompassing both cognitive and affective components [3].

However, contemporary ethics education in healthcare predominantly emphasizes principlist approaches, focusing on abstract ethical theories and decision-making frameworks [4]. While such principle-based instruction provides essential foundational knowledge, emerging research suggests these approaches alone are insufficient for developing practitioners who can recognize ethical concerns in complex, real-world clinical contexts [5]. The gap between knowing ethical principles and recognizing when they apply in practice remains poorly understood, particularly in healthcare professional education. Team-based learning and interactive pedagogies have shown promise in bridging this theory-practice gap [6], yet fundamental questions persist about what contextual factors activate ethical sensitivity in situ.

A critical contextual factor that may influence whether clinicians recognize ethical issues is moral intensity, defined as the extent to which an ethical issue engages a person’s values and elicits emotional salience [7]. Moral intensity operates across multiple dimensions: magnitude of consequences, social consensus, probability of effect, temporal immediacy, proximity to the ethical issue, and concentration of effect. Research in business ethics and organizational behavior has demonstrated that higher moral intensity significantly increases the likelihood of ethical cognition and decision-making, influencing both moral courage and decision-making abilities [8, 9]. Yet despite decades of moral intensity research in non-healthcare contexts, limited empirical investigation has examined whether and how moral intensity influences distinct components of ethical sensitivity in healthcare professional education.

While vignette-based assessments such as the MIEST provide a structured and standardised approach to examining ethical sensitivity, they capture responses to hypothetical scenarios rather than real-time clinical behaviour. As such, they are best understood as assessing articulated and reflective components of ethical sensitivity, rather than the full range of perceptual, relational, and contextually embedded processes that occur in clinical practice.

Furthermore, ethical sensitivity itself appears to be multidimensional, encompassing both cognitive-analytical capacities, such as abstract recognition of ethical principles, and perceptual-affective capacities, such as intuitive, emotionally informed detection of ethical salience in specific contexts [10]. Contemporary bioethicists have drawn on Aristotle’s classical distinction between logos (rational thought), ethos (character), and pathos (emotional resonance) to argue that ethical practice requires integration of these dimensions [11]. Moral imagination, the capacity to recognize and envision the moral consequences of decisions, has also been identified as critical for developing ethical sensitivity in both biomedical researchers and healthcare professionals [12]. This theoretical framework suggests that contexts characterized by higher moral intensity (by definition more emotionally engaging and value-laden) may be associated with greater engagement of the perceptual-affective components of ethical sensitivity while leaving analytical principle-recognition relatively unchanged.

The therapeutic sciences, encompassing audiology, speech-language pathology, physiotherapy and occupational therapy, remain substantially understudied in healthcare ethics research compared to medicine and nursing. These professions share critical characteristics that make ethics education particularly important: they involve intimate clinical relationships with vulnerable and dependent populations, require substantial autonomous clinical decision-making, and increasingly face emerging ethical challenges related to evolving technology, health equity, scope of practice expansion, and interprofessional collaboration [13]. Physical therapists, in particular, have demonstrated both high ethical sensitivity and moral sensitivity, yet factors influencing their ethical development require further investigation [14]. Understanding factors that enhance ethical sensitivity in therapeutic sciences clinicians is therefore both timely and essential for the profession.

The current study addresses this significant gap by examining whether moral intensity, operationalized as expert-rated characteristics of ethical scenarios, is differentially associated with components of ethical sensitivity in pre-service therapeutic sciences clinicians. We hypothesized that moral intensity would show selective associations with ethical sensitivity components, being more strongly related to ethical sensitivity skills (perceptual-affective detection of ethical salience) than to ethical principle recognition (analytical, knowledge-based capability). This hypothesis draws on cognitive and neuroscience research demonstrating that emotional arousal and value engagement enhance perceptual attention and pattern recognition [15], with moral intensity potentially activating System 1 (intuitive) rather than System 2 (deliberative) cognitive processes [16]. If supported, such findings would have direct implications for reconceptualizing ethical sensitivity as multidimensional and for designing ethics curricula that intentionally engage both rational and emotional dimensions of ethical awareness. While previous work using this dataset has focused on instrument validation, the present study examines a distinct research question relating to the role of moral intensity in ethical sensitivity.

## Methods

This study reports a secondary exploratory analysis of data collected during the development and validation of the MIEST. This secondary analysis falls within the scope of the original ethical approval and participant consent, which included permission for anonymised data to be used in future research related to ethical sensitivity in healthcare education. The original data collection was approved by the University of Pretoria Faculty of Health Sciences Research Ethics Committee (Reference: HUM011/2015). The current analysis examines associations between expert-rated moral intensity and components of ethical sensitivity that were not addressed in prior publications. While the original dataset was used for instrument development and validation [17], with findings also reported in subsequent publications [18], the present study represents a secondary analysis addressing a distinct research question not examined in prior work.

### Study design and participants

A cross-sectional design was employed. Participants were 120 final-year pre-service clinicians enrolled in the Department of Health and Rehabilitation Sciences at a South African university. The sample included students from four therapeutic sciences programs: audiology (*n* = 28), speech-language pathology (*n* = 32), physiotherapy (*n* = 31), and occupational therapy (*n* = 29). Final-year students were selected because they have completed the majority of their professional ethics education and clinical training, allowing for a more integrated assessment of ethical sensitivity as it develops within healthcare education. At this stage of training, students are expected to engage with both theoretical and applied dimensions of ethical practice, making them an appropriate group for examining associations between scenario characteristics and ethical sensitivity responses. Participants were drawn from multiple therapeutic disciplines because the MIEST was designed as a discipline-neutral instrument; accordingly, responses were aggregated across disciplines in line with the vignette-level analytic focus on scenario characteristics rather than group differences.

Inclusion criteria were: (1) enrolled as a final-year student in one of the four therapeutic sciences programs, (2) completed at least 70% of the professional curriculum including ethics coursework, and (3) able to provide informed consent. Exclusion criteria were: (1) not a native or fluent English speaker (given the English-language vignettes), and (2) previous research participation in MIEST validation studies.

### Instrument: MIEST

The MIEST is a scenario-based assessment tool comprising 12 ethical vignettes set in therapeutic healthcare contexts. Each vignette (approximately 150–200 words) describes a realistic ethical scenario requiring clinical decision-making. Scenarios were developed to represent diverse ethical issues common in therapeutic practice (e.g., confidentiality breaches, conflicts of interest, resource allocation, cultural competence challenges, professional boundary violations).

For each vignette, participants were asked to: (1) identify which ethical principles (from a list of 10 principles: autonomy, beneficence, non-maleficence, justice, fidelity, veracity, confidentiality, professional competence, respect for persons, accountability) were relevant to the scenario—the ethical principle recognition measure; and (2) describe what ethical concerns or dilemmas they perceived in the scenario—the ethical sensitivity skills measure.

Moral Intensity Assessment: Following data collection, two bioethics experts independently rated each of the 12 vignettes across the six dimensions of moral intensity as defined by Jones [7]: magnitude of consequences, social consensus, probability of effect, temporal immediacy, proximity and concentration of effect. Each dimension was rated on a 5-point scale (1 = very low to 5 = very high), resulting in six dimension-specific ratings for each vignette from each rater.

To support consistency in scoring, the two raters engaged in a calibration exercise prior to independent rating, during which scoring criteria and interpretation of each dimension were discussed and aligned. A composite moral intensity score for each vignette was calculated by summing ratings across the six dimensions (range 6–30) and averaging across the raters to produce a final vignette-level score.

Inter-rater agreement was assessed using the mean absolute difference between the two raters, which was 1.08 points per vignette on the 6–30 scale, indicating a high level of agreement relative to the total possible range. This level of agreement suggests an acceptable level of inter-rater agreement for an exploratory analysis. Minor discrepancies (typically 1–2 points) were resolved through discussion to reach consensus scores, which were used in the final analysis. Given the limited number of vignettes (*n* = 12), formal reliability statistics such as intraclass correlation coefficients were not calculated, as these would not yield stable estimates.

### Outcome measures

Ethical Principle Recognition: Participants’ responses to the ethical principle identification question were scored for accuracy. For each vignette, a set of ethical principles considered relevant to the scenario had been established during the original MIEST development process. A participant response was scored as correct if it identified one or more of the ethical principles included in this predefined expert-derived answer set. For each vignette, the proportion of participants correctly identifying a relevant ethical principle was calculated and expressed as a percentage.

Ethical Sensitivity Skills: Responses to the open-ended ethical concern question were scored using a rubric assessing: (1) the number of distinct ethical concerns identified, (2) the sophistication of concern articulation (superficial vs. nuanced), and (3) the extent to which contextual features of the scenario were incorporated into the response. Responses demonstrating recognition of relevant ethical concerns, consideration of stakeholder perspectives, and acknowledgement of contextual influences were classified as meeting the criteria for correct ethical sensitivity skill identification. Responses were independently scored by two raters (κ = 0.81, indicating good inter-rater reliability) with disagreements resolved by consensus. All ethical sensitivity outcomes were first calculated at the participant level and then aggregated at the vignette level to produce a single value per vignette. For each vignette, percentage scores were calculated by dividing the number of participants who met the criteria for correct identification by the total number of participants (*N* = 120) and multiplying by 100. Ethical principle recognition percentages reflect the proportion of participants correctly identifying a relevant ethical principle, whereas ethical sensitivity skill percentages reflect the proportion of participants meeting the rubric criteria for ethical concern recognition, contextual interpretation, and stakeholder awareness. These percentage values were used as the primary outcome measures in the vignette-level analysis.

### Data analysis

Statistical analyses were conducted using IBM SPSS Statistics (Version 29). All inferential analyses were performed at the level of the vignette (*n* = 12). Although data were collected from 120 participants, these responses were aggregated to generate a single value per vignette, and thus the effective sample size for statistical analysis was 12. For each vignette, moral intensity was operationalised as a composite score (range 6–30), derived by summing ratings across the six dimensions of Jones’s framework and averaging across the two raters [7]. Ethical sensitivity outcomes were aggregated across participants to produce vignette-level variables, including (a) the percentage of participants correctly identifying the ethical principle, (b) the percentage correctly identifying ethical sensitivity skills, and (c) the mean total MIEST score (0–8).

Pearson’s product–moment correlation coefficients were calculated to examine associations between moral intensity scores and each ethical sensitivity outcome. This approach reflects the study’s focus on relationships between scenario characteristics and aggregated response patterns, rather than individual-level variation.

Exploratory subsidiary analyses examined whether specific moral intensity dimensions (magnitude of consequences, social consensus, probability of effect, temporal immediacy, proximity, and concentration of effect) showed differential associations with outcomes. Given the limited sample size of vignettes (*n* = 12), these dimension-level analyses are presented as descriptive findings to inform future research directions. See Supplementary Table S1 for complete dimension-level ratings. Because the analysis was conducted at the vignette level, the results reflect associations between characteristics of scenarios and aggregated responses, and do not permit inference about relationships at the level of individual participants. Because moral intensity was invariant within each vignette, it was not statistically appropriate to examine correlations at the individual participant level, as this would violate assumptions of independence and potentially lead to misleading inferences.

## Results

### Descriptive statistics

All results are reported at the vignette level (*n* = 12), with values representing aggregated responses across 120 participants and derived directly from vignette-level values presented in Table 1. Consequently, all inferential findings are based on 12 vignette-level observations. All twelve vignettes demonstrated variability in moral intensity and ethical sensitivity outcomes. Moral intensity scores ranged from 20 to 28 on a possible scale of 6–30, indicating moderate to high perceived ethical urgency across vignettes (M = 24.2, SD = 2.4).

**Table 1:** Moral intensity and ethical sensitivity outcomes across clinical vignettes

Vignette number	Moral intensity score (6–30)	Ethical principle correctly identified (%)	Ethical sensitivity skill correctly identified (%)	Overall ethical sensitivity (%)¹
1	20	79.2	60.2	69.7
2	26	94.2	94.7	94.5
3	23	77.3	35.5	56.4
4	27	28.5	95.5	62.0
5	22	80.0	60.2	70.1
6	22	59.3	55.0	57.2
7	25	94.5	84.8	89.7
8	22	74.5	65.0	69.8
9	28	90.0	95.5	92.8
10	25	93.5	74.2	83.9
11	26	45.2	89.8	67.5
12	24	61.7	69.3	65.5

Moral intensity scores were derived using Jones’s six-dimension framework [7]. Ethical sensitivity outcomes are aggregated at the vignette level across all participants (*N* = 120)

¹ Overall ethical sensitivity (%) represents the mean of ethical principle recognition (%) and ethical sensitivity skill identification (%) for each vignette

Across vignettes, the proportion of participants who correctly identified the relevant ethical principle ranged from 28.5% to 94.5% (M = 73.2%, SD = 20.9%). Correct identification of ethical sensitivity skills ranged from 35.5% to 95.5% (M = 73.3%, SD = 19.2%). Overall ethical sensitivity index—calculated as the mean of principle recognition and skill identification percentages—ranged from 56.4% to 94.5% (M = 73.3%, SD = 13.5%).

Complete vignette-level descriptive statistics, including moral intensity scores, ethical principle recognition percentages, ethical sensitivity skill identification percentages, and overall ethical sensitivity metrics for all 12 vignettes, are presented in Table 1. Detailed dimension-level ratings for each of the six moral intensity dimensions (magnitude of consequences, social consensus, probability of effect, temporal immediacy, proximity, and concentration of effect) are provided in Supplementary Table S1.

#### Main findings

Moral Intensity and Ethical Principle Recognition: No statistically significant correlation was observed between moral intensity and percentage of participants correctly identifying ethical principles (*r* = -0.10, *p* = 0.755). Recognition of ethical principles was largely independent of the perceived emotional or contextual intensity of the scenario, suggesting that ethical principle identification reflects cognitive processes relatively insulated from contextual variations in moral salience.

**Fig. 1:**
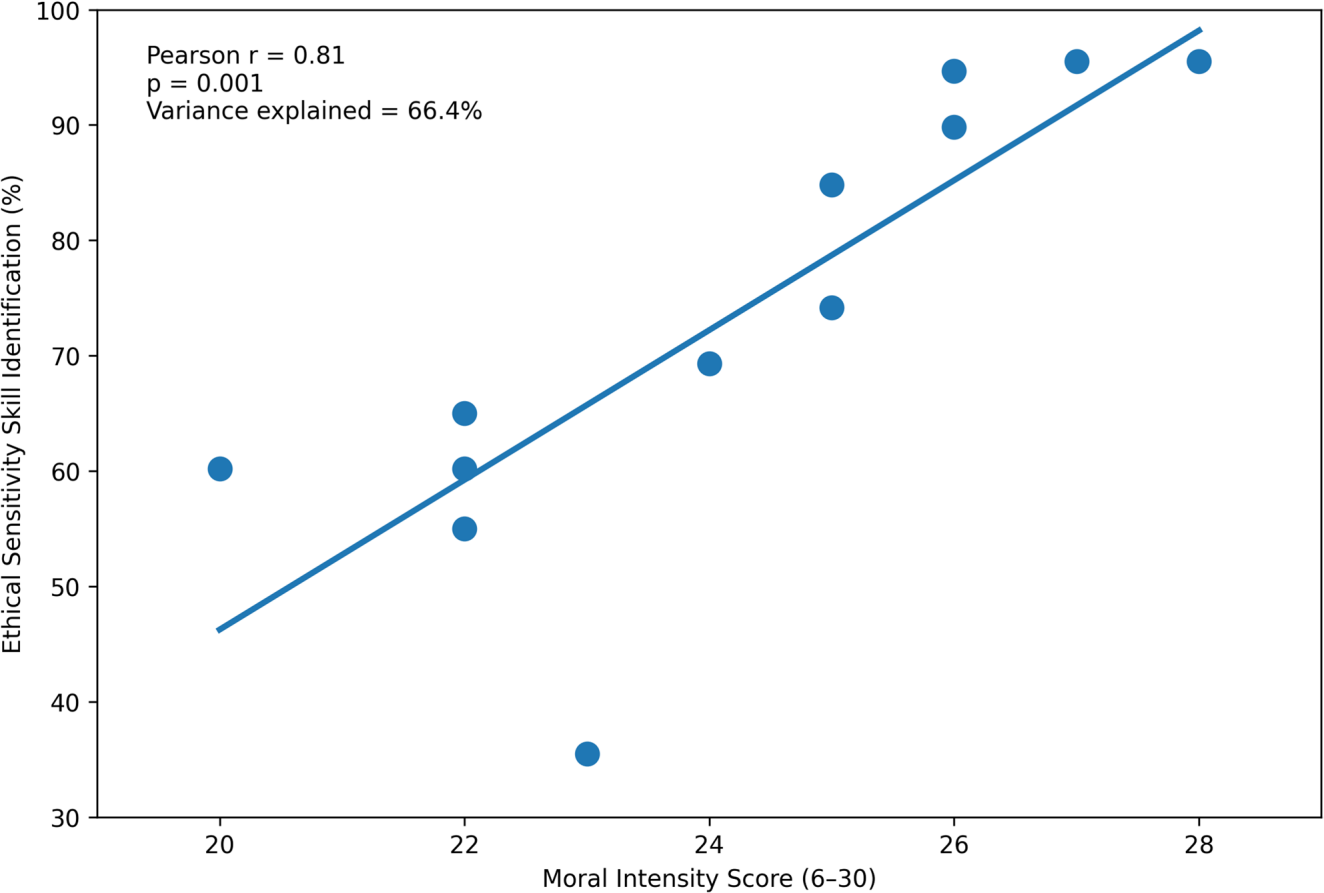
Relationship between moral intensity and ethical sensitivity skill identification (%). Scatter plot showing the relationship between vignette-level moral intensity scores (range 6–30) and ethical sensitivity skill identification (%) across 12 clinical vignettes. Each point represents one vignette. The fitted line represents the linear association between the variables (Pearson *r* = 0.81, *p* = 0.001)

Moral Intensity and Ethical Sensitivity Skill Identification: A statistically significant positive correlation was found between moral intensity and ethical sensitivity skill identification (*r* = 0.81, *p* = 0.001). Vignettes rated as higher in moral intensity were significantly more likely to elicit correct identification of ethical sensitivity skills including perspective-taking, contextual interpretation, and morally appropriate action. This association corresponds to 66.4% shared variance between the variables (r² = 0.664). This was the only statistically significant relationship found between moral intensity and components of ethical sensitivity. See Fig. 1 for visualization of this significant association.

Moral Intensity and Overall Ethical Sensitivity: An exploratory analysis examined the relationship between moral intensity and overall ethical sensitivity. A moderate positive correlation was observed (*r* = 0.50, *p* = 0.098), though this did not reach statistical significance at the conventional alpha = 0.05 threshold. The medium effect size suggests a possible relationship that warrants further investigation with larger sample sizes. 

#### Exploratory dimension-level analyses

Preliminary examination of specific moral intensity dimensions suggests that concentration of effect and magnitude of consequences showed the strongest numerical correlations with ethical sensitivity skills, though these dimension-level findings are exploratory and should be interpreted cautiously given the small number of vignettes. Complete dimension-level data is provided in Supplementary Table S1.

## Discussion

The findings should be interpreted within the constraints of a small vignette-level sample (*n* = 12) and are therefore exploratory in nature. This exploratory vignette-level study examined whether moral intensity is differentially associated with components of ethical sensitivity among pre-service clinicians in the therapeutic sciences. The findings indicate a selective relationship: moral intensity was strongly associated with ethical sensitivity skill identification but not with ethical principle recognition. These findings provide preliminary empirical support for ethical sensitivity as a multidimensional construct; however, the results should be interpreted within the constraints of an exploratory vignette-level analysis based on 12 observations.Findings suggest that contextual features of ethical scenarios may differentially engage perceptual–affective versus cognitive–analytical components of moral functioning. Consequently, the findings reflect associations at the level of scenario characteristics rather than direct measures of ethical sensitivity as enacted in clinical practice.

### Moral intensity and ethical principle recognition

No statistically significant association was observed between moral intensity and ethical principle recognition. This suggests that participants’ ability to identify relevant ethical principles was relatively stable across scenarios with varying degrees of ethical urgency or emotional salience. Ethical principle recognition reflects a largely knowledge-based and analytic process, typically developed through formal ethics education and professional codes [4, 19]. As such, it may be less sensitive to contextual variation in moral intensity.

This finding is consistent with prior work indicating that principle-based ethical knowledge does not necessarily translate into heightened ethical awareness in practice [5]. Individuals may correctly identify abstract principles even in low-intensity or hypothetical cases, without necessarily engaging with the relational or contextual complexity of the situation. The absence of an association therefore supports the conceptual distinction between ethical principle recognition and broader ethical sensitivity.

### Moral intensity and ethical sensitivity skills

In contrast, a strong positive association was observed between moral intensity and ethical sensitivity. Vignettes rated as higher in moral intensity were more likely to elicit correct ethical sensitivity skill identification involving stakeholder perspectives, contextual interpretation, and morally appropriate action. These skills correspond to perceptual–affective components of ethical sensitivity rather than rule-based reasoning alone [3, 10].

Moral intensity, as conceptualised by Jones [7], captures features of situations that heighten moral attention, including proximity and the perceived magnitude or probability of consequences. Such features may amplify emotional engagement and value salience and may be associated with increased detection of ethical concerns. This interpretation is consistent with cognitive and moral psychology literature suggesting that emotionally salient contexts enhance perceptual attention and intuitive pattern recognition [15, 16]. However, given the exploratory design and limited vignette-level sample, this explanation should be regarded as a possible interpretation rather than a direct test of these theoretical mechanisms.

Importantly, this association does not suggest that higher moral intensity improves ethical competence overall. Rather, it indicates that morally intense scenarios may preferentially activate ethical sensitivity skills related to noticing and interpreting ethical salience. This distinction is critical for understanding why clinicians may possess ethical knowledge yet fail to recognise ethical issues in everyday practice.

### Overall ethical sensitivity scores

Moral intensity was not significantly associated with overall ethical sensitivity scores. Given that the composite score aggregates both ethical principle recognition and ethical sensitivity skills, this null finding is not unexpected. Aggregation may obscure divergent patterns across components, particularly when one component (ethical principle recognition) is relatively invariant across contexts. This result reinforces the importance of avoiding unitary interpretations of ethical sensitivity and supports analytic approaches that examine its components separately.

### Methodological considerations

This study adopted a vignette-level analytic approach, conceptualising moral intensity as a property of the scenario rather than of individual participants. Ethical sensitivity outcomes were therefore aggregated across participants for each vignette. Although participant responses were nested within vignettes, multilevel modelling was not applied due to the limited number of higher-level units (*n* = 12) and the invariance of moral intensity within each vignette. Under these conditions, multilevel models would not have produced stable or interpretable estimates.

While moral intensity is ideally assessed through participant perception, expert-based ratings were used to ensure consistency across vignettes in this exploratory analysis. Future studies should directly compare these expert ratings with participant-perceived moral intensity to examine whether subjective experience modifies these associations.

Vignette-based assessment methods are inherently limited in their ability to capture non-verbal, intuitive, and contextually embedded aspects of ethical sensitivity that emerge in real-time clinical interactions. As such, the present findings should be interpreted as reflecting structured ethical perception rather than fully enacted clinical ethical sensitivity. This design constraint precluded examination of individual-level associations between moral intensity and ethical sensitivity, and findings should therefore be interpreted strictly at the level of vignette characteristics. Because the analysis was conducted at the vignette level, discipline-specific variation could not be examined, and the findings represent aggregated response patterns across the included therapeutic fields.

### Implications for ethics education

The findings have important implications for ethics education in healthcare. First, they suggest that teaching ethical principles alone may be insufficient for developing ethical sensitivity in practice. While principle knowledge is necessary, it may not enhance clinicians’ ability to notice ethical concerns unless accompanied by contextual and emotionally engaging learning experiences.

Second, the results support the use of pedagogical approaches that emphasise rich, realistic scenarios with higher moral intensity, such as case-based learning, debate-based ethics education, and simulation [6, 18, 20]. Such approaches may be particularly effective in cultivating ethical sensitivity skills that rely on contextual interpretation and moral imagination [12].

Finally, the selective association observed in this study highlights the value of explicitly distinguishing between components of ethical sensitivity in both assessment and curriculum design. Ethics education that intentionally targets perceptual–affective dimensions may better prepare clinicians to recognise ethical challenges in complex clinical environments [20, 21].

Given the exploratory design and limited number of vignette-level observations, these findings should be interpreted as hypothesis-generating rather than confirmatory.

### Limitations and future directions

Several limitations should be acknowledged. First, the effective sample size for inferential analysis was limited to 12 vignette-level observations, which constrains statistical power and increases sensitivity to outliers [22]. Second, moral intensity was assessed via expert ratings rather than participant self-report. Third, the study involved a secondary analysis of data collected for instrument validation, limiting control over vignette construction and moral intensity distribution. Fourth, the sample comprised final-year pre-service clinicians within a South African healthcare education context. The findings are therefore most appropriately interpreted within this context and may not generalise to experienced clinicians, earlier stages of training, or other professional domains. Additionally, the aggregation of participants across disciplines precludes conclusions about discipline-specific patterns of ethical sensitivity.

These limitations indicate that the findings should be interpreted as exploratory and hypothesis-generating. Future research should employ prospective designs that experimentally manipulate moral intensity, include participant-rated intensity measures, and examine longitudinal development of ethical sensitivity across training stages and professional groups.

## Conclusion

This exploratory study provides preliminary evidence that moral intensity may be differentially associated with specific components of ethical sensitivity. Given the vignette-level design and limited number of observations, these findings should be viewed as hypothesis-generating and require confirmation in larger studies. The findings reinforce ethical sensitivity as a multidimensional construct and demonstrate that contextual and affective features of ethical scenarios may differentially engage components of moral functioning. By distinguishing between ethical sensitivity components, this work contributes to empirical ethics research and informs the design of ethics education in the therapeutic sciences and broader healthcare contexts. 

## Supplementary Information

Supplementary Material 1.

## Data Availability

Data supporting these findings are available within the article and its additional files. Supplementary Table S1 provides vignette-level descriptive statistics. Raw data are available from the corresponding author upon request, subject to ethical and privacy restrictions.
