# Supplementary material for: Moral intensity and ethical sensitivity in pre-service therapeutic sciences clinicians: a vignette-level analysis of ethical principle recognition and ethical sensitivity skills
Source: BMC Med Ethics. 2026 Jun 12;27:169. doi: 10.1186/s12910-026-01521-4 (PMC13483608; doi:10.1186/s12910-026-01521-4)
Supplement: Supplementary file 1 [file 12910_2026_1521_MOESM1_ESM.docx]

**Supplementary table s1: Moral Intensity Dimension Ratings Across Clinical Vignettes**

# This table presents comprehensive vignette-level results for all 12 vignettes, including: moral intensity composite scores (range 6-30), ethical principle recognition percentages, ethical sensitivity skill identification percentages, and overall ethical sensitivity index percentages. This table corresponds to the complete results data referenced throughout the manuscript.

| **Vignette** | **Magnitude of consequences** | **Social consensus** | **Probability of effect** | **Temporal immediacy** | **Proximity** | **Concentration of effect** | **Total score** | **Total score (%)** |
| --- | --- | --- | --- | --- | --- | --- | --- | --- |
| 1 | 2 | 4 | 5 | 4 | 4 | 1 | 20 | 67 |
| 2 | 4 | 5 | 4 | 4 | 5 | 4 | 26 | 87 |
| 3 | 4 | 4 | 4 | 3 | 4 | 4 | 23 | 77 |
| 4 | 5 | 5 | 4 | 3 | 5 | 5 | 27 | 90 |
| 5 | 4 | 5 | 3 | 3 | 4 | 3 | 22 | 73 |
| 6 | 3 | 4 | 4 | 3 | 4 | 4 | 22 | 73 |
| 7 | 4 | 4 | 4 | 4 | 5 | 4 | 25 | 83 |
| 8 | 3 | 4 | 4 | 3 | 4 | 4 | 22 | 73 |
| 9 | 5 | 5 | 5 | 4 | 4 | 5 | 28 | 93 |
| 10 | 4 | 5 | 4 | 3 | 5 | 4 | 25 | 83 |
| 11 | 4 | 5 | 5 | 3 | 4 | 5 | 26 | 87 |
| 12 | 4 | 5 | 4 | 3 | 4 | 4 | 24 | 80 |

*Note.* Moral intensity dimensions were rated using Jones’s six-dimension framework [7] on a 5-point Likert scale (1 = very low, 5 = very high). Total scores represent the sum of all six dimensions (possible range = 6–30). Percentage scores represent the total score expressed as a proportion of the maximum possible score (30), multiplied by 100. Values are rounded to the nearest whole number. Dimension-level ratings are provided for descriptive transparency.
